# Small Molecule BRD4 Inhibitors Apabetalone and JQ1 Rescues Endothelial Cells Dysfunction, Protects Monolayer Integrity and Reduces Midkine Expression

**DOI:** 10.3390/molecules27217453

**Published:** 2022-11-02

**Authors:** Sidra Shahid, Marlena Pantakani, Lutz Binder, Andreas Fischer, Krishna Pantakani, Abdul R. Asif

**Affiliations:** 1 Institute for Clinical Chemistry, University Medical Centre Goettingen, 37075 Goettingen, Germany; 2 German Centre for Cardiovascular Research (DZHK), Partner Site Goettingen, 37075 Goettingen, Germany; 3 Division Vascular Signaling and Cancer, German Cancer Research Center (DKFZ), 69120 Heidelberg, Germany

**Keywords:** BRD4, endothelial cells, inflammation, tight junction, midkine, endothelial dysfunction

## Abstract

NF-κB signaling is a key regulator of inflammation and atherosclerosis. NF-κB cooperates with bromodomain-containing protein 4 (BRD4), a transcriptional and epigenetic regulator, in endothelial inflammation. This study aimed to investigate whether BRD4 inhibition would prevent the proinflammatory response towards TNF-α in endothelial cells. We used TNF-α treatment of human umbilical cord-derived vascular endothelial cells to create an in vitro inflammatory model system. Two small molecule inhibitors of BRD4—namely, RVX208 (Apabetalone), which is in clinical trials for the treatment of atherosclerosis, and JQ1—were used to analyze the effect of BRD4 inhibition on endothelial inflammation and barrier integrity. BRD4 inhibition reduced the expression of proinflammatory markers such as *SELE*, *VCAM-I*, and *IL6* in endothelial cells and prevented TNF-α-induced endothelial tight junction hyperpermeability. Endothelial inflammation was associated with increased expression of the heparin-binding growth factor midkine. BRD4 inhibition reduced midkine expression and normalized endothelial permeability upon TNF-α treatment. In conclusion, we identified that TNF-α increased midkine expression and compromised tight junction integrity in endothelial cells, which was preventable by pharmacological BRD4 inhibition.

## 1. Introduction

Atherosclerosis is a chronic inflammatory disease in which lipids and fibrous material accumulate in arterial walls. Inflammation of endothelial cells (ECs) leads to the initiation and progression of plaque formation [1]. Multiple factors, including hyperglycemia, dyslipidemia, disturbed blood flow, and hypertension, exert continuous stress on ECs, causing ECs dysfunction and, finally, atherosclerosis development [2,3]. Endothelial dysfunction is characterized by increased reactive oxygen species (ROS) production and apoptotic activity of ECs, reduced endothelial specific nitric oxide synthase (eNOS) expression, reduced nitric oxide production, and augmented permeability of the endothelial barrier [4,5].

Nuclear transcription factor κB (NF-κB) is a master regulator of inflammation. Cytokines like interleukin-1β and tumor necrosis factor-α (TNF-α) activate NF-κB and protein kinase C (PKC) pathways [6]. Subsequently, IκB-kinase (IKK) phosphorylates IκB-α, which gets ubiquitinated and degraded by the proteasome. NF-κB is then translocated to the nucleus where it induces the transcription of multiple proinflammatory genes, such as cytokines (IL6, IL8), TNF-α, and leukocyte adhesion molecules (E-SELE, ICAM-1, VCAM-1) [7].

Bromodomain-containing protein 4 (BRD4), an epigenetic regulator, is a member of the BET (Bromodomain and extra terminal domain) tandem bromodomain-containing family [8]. In a healthy cell state, BRD4 regulates multiple cellular activities, including cell cycle, spermatogenesis, and DNA damage repair. BRD4 binds to acetylated chromatin and further acetylates the core of histones at H3K122 residues, which leads to nucleosome disassembly [8,9].

In ECs, BRD4 expression is higher than other BET protein family members. BRD4 has two naturally existing splice variants/isoforms known as BRD4 long (BRD4-L) and BRD4 short (BRD4-S) [10]. The protein structures of BRD4-L and BRD4-S proteins are similar at the N-terminal region, except for the last three amino acids, while BRD4-L has an extra C terminal domain which is absent in the BRD4-S isoform. BRD4-S isoform exhibits higher affinity to bind with chromatin as compared to BRD4-L [11]. BRD4 isoforms play distinct roles in breast cancer progression, where BRD4-S enhances cancer metastasis while the BRD4-L isoform reduces metastasis [10,12,13]. Similarly, BRD4 isoforms are known for their opposite roles in DNA damage repair [11].

In ECs, BRD4 is responsible for maintaining basal cellular functions by interacting at super-enhancers (SEs) of genes responsible for maintaining a basal cell state. Translocation of NF-κB from the cytoplasm to nucleus under inflammatory conditions leads to enhanced proinflammatory gene expression [5]. BRD4 redistributes on chromatin upon inflammation and, together with NF-κB, binds to SEs of proinflammatory genes [14]. BRD4 acts as a coactivator of the RelA subunit of NF-kB by binding to its acetylated lysine-310, and this interaction is necessary for the initiation of an inflammatory response [15]. BRD4 and NF-κB interaction at SEs of proinflammatory genes leads to RNA polymerase II phosphorylation, which ensures the transcription elongation of target genes, including adhesion molecules and inflammatory cytokines [13,15]. Studies using chromatin immunoprecipitation (ChIP) and parallel DNA sequencing (ChIP-seq) in ECs showed that RelA and BRD4 were enriched on promoter and SE regions of 271 genes, including SELE and VCAM1, after exposure to proinflammatory stimuli [6,14]. As such, BRD4 is an interesting target to inhibit endothelial inflammation and subsequent dysfunction.

Among different cytokines, Midkine, a small growth factor protein 13 kDa in size, is an interesting target that was reported to be highly expressed in inflammatory kidney diseases and atherosclerosis. Midkine expression is also high in atherosclerotic lesions in mice models, and midkine^−/−^ mice showed reduced recruitment and transmigration of inflammatory cells [16,17]. Previously reported studies have shown that elevated midkine expression is associated with increased endothelial permeability [18].

Therefore, in this study, the role of BRD4 was investigated in a model system of endothelial inflammation to better understand whether pharmacological BRD4 inhibition with apabetalone and JQ1 might be sufficient to prevent endothelial monolayer disturbance during the inflammatory response.

## 2. Materials and Methods

### 2.1. Cell Culture

Primary cultures of human umbilical cord-derived venous vascular endothelial cells (HUVECs, purchased from PromoCell, Heidelberg, Germany), were used for all in vitro experiments under sterile conditions. HUVECs were grown in endothelial cells basal growth medium containing 2% fetal calf serum, 0.1 ng/mL epidermal growth factor, 1 ng/mL fibroblast growth factor (PromoCell), and 1% Penicillin/Streptomycin (Invitrogen, Waltham, MA, USA) in a humidified incubator with 5% CO_2_ at 37 °C until passage 6 and experiments were performed at passage 7. Gelatin (0.2%) coated flasks/plates were used for cell culture. 5 × 10^4^ cells/cm^2^ were used for all treatments. To create an inflammatory in vitro model, HUVECs were treated with 20 ng/mL of human recombinant TNF-α (Peprotech, Hamburg, Germany). Different treatment durations and concentrations of BRD4 inhibitors were used for optimization. The final concentrations used were 500 nM JQ1 (ApexBio technologies, Houston, TX, USA) or 60 μM RVX208 (ApexBio technologies) for 12 h. Control cells were treated with an equal volume of DMSO. For treatments, cells were first incubated with inhibitors for 12 h. Later, after washing with PBS, a fresh medium with TNF-α was added for the indicated time to create an inflammation model.

### 2.2. Immunofluorescence Staining of HUVECs

HUVECs were grown on 0.2% gelatin coated small coverslips. Cells were washed with Dulbecco’s phosphate-buffered saline (DPBS) after the completion of the experiment. 4% paraformaldehyde (PFA) was used for fixation of cells followed by 2 times washing with DPBS for 10 min each. Next, 50 mM of ammonium chloride (NH_4_Cl) in DPBS was used for blocking for 10 min at room temperature (RT). Cell permeabilization was done with 0.2% Triton X-100 (Sigma, St. Louis, MO, USA) in DPBS buffer (3 times 4 min each), followed by incubation with anti-NF-κB antibody (Cell Signaling, Leiden, The Netherlands. cat no, mAb #8242), 1:100 dilution in permeabilization buffer at RT for 1 h. Afterward, cells were again washed 3 times for 4 min each with 0.2% Triton X-100 in DPBS. Cells were incubated with a secondary antibody anti-rabbit IgG conjugated with Alexa 488 (Life Technologies, Darmstadt, Germany), at 1:300 for 1 h at RT. Excessive antibody was removed by washing again with 0.2% Triton X-100 in DPBS (3 times 4 min). Finally, cells were mounted with 4′,6-diamidino-2phenylindole, dihydrochloride (DAPI) (Vector Laboratories, Newark, NJ, USA). Axiovert 200M confocal Microscopy (Carl Zeiss, Jena, Germany) was used to visualize the fluorescent cells.

### 2.3. RNA Extraction from Cells

Trizol (Invitrogen) was used for total RNA extraction according to the manufacturer’s instructions. Briefly, cells were treated, washed twice, and then harvested. 500 μL per 10 cm^2^ of Trizol reagent was added and cells were homogenized by vortexing and chloroform/isopropanol extraction method was used for total RNA precipitation. RNA was washed with 70% ethanol and air dried, then dissolved in H_2_O treated with diethylpyrocarbonate (DEPC) (Sigma) and stored at −80 °C. NanoDrop 2000C (PeqLab, ThermoFischer Scientific, Darmstadt, Germany) was used for RNA quantification.

### 2.4. cDNA Synthesis and qRT-PCR

Before cDNA preparation, DNase digestion was performed on total RNA. cDNA was prepared by SuperScript™ IV First-Strand Synthesis System (Invitrogen) following the manufacturer’s protocol. Briefly, 2 μg of RNA was incubated with 1 μL of 10× DNase reaction buffer (Sigma) and 1 μL of amplification grade DNase enzyme (Sigma) in 8 μL DEPC water for 15 min at RT. The reaction was inhibited with 1 μL of Stop solution (Sigma) and heated at 70 °C for 5 min. Then 0.5 μMol/L each dNTP (Roche), 0.15 μM Primer p(dT)15 for cDNA synthesis (Roche), 1X Reverse Transcriptase buffer (Invitrogen), 1 U/μL RNase inhibitor (Promega), and 13.3U M-MLV-RT (Invitrogen) enzyme were added and incubated at 42 °C for 1 h. The reaction mix was heated at 70 °C for 10 min to inactivate the enzymes. cDNA was stored at −20 °C. Primers were designed by using Primer 3 web input and the primer sequence was confirmed by the UCSC Genome browser (Supplementary Table S1). Primers were first run in conventional PCR machines and the product was loaded on 1.5% agarose gel. The product size was confirmed (data not shown). PCR reactions were run and analyzed on LightCycler 480 software SW 1.5.1 (Roche). The comparative Ct method (2^−∆∆Ct^) was used to measure relative change in mRNA expression [19].

### 2.5. Cell Viability Assay

Trypan blue dye exclusion assay method was used to determine the cell viability after each experiment. Briefly, cells were treated, trypsinized, and resuspended in 1 mL of cell culture media. Suspended cells were mixed with media and 0.5% trypan blue dye in a 1:4:5 ratio. 10 μL of the mixture was loaded onto a haemocytometer. Viable cells (colorless) and nonviable cells (blue colored) were counted, and the percentage of viable cells was calculated.

### 2.6. Protein Isolation and Western Blot

HUVECs were grown, treated as discussed above, washed with DPBS, and trypsinized to collect. DPBS washed cells pellets were suspended using lysis buffer (Cell Signaling), Phospho-stop phosphatase inhibitor (Sigma), ethylenediaminetetraacetic acid (EDTA)-free protease inhibitor cocktail (Sigma), and 1 mM phenylmethylsulfonyl fluoride (PMSF) were added fresh. Lysates were centrifuged at 14,000 rpm for 15 min and clear supernatants (total proteins) were separated. Bradford method (Bio-Rad) was used for protein quantification. Proteins (35 μg) were resolved on 4–12% Bis-Tris sodium dodecyl sulfate (SDS) polyacrylamide gels (Thermo Fischer Scientific), together with molecular weight standards, Proteins were transferred onto a nitrocellulose membrane of 0.4 μM pore size (Millipore). To avoid nonspecific binding, membranes were blocked in blocking buffer: 5% milk in Tris-buffered saline, containing 50 mM Tris-HCl, 200 mM NaCl, and 0.1% Tween-20 (TBST). Blocked membranes were incubated with indicated primary antibodies namely, anti-BRD4-antibody (Abcam #ab84776) in 1:1000 dilution, anti-β-actin-antibody (Sigma #A5316) in 1:10,000 dilution and anti-midkine-antibody (Abcam #ab36038) in 1:500 dilution overnight at 4 °C in blocking buffer. The next day, membranes were washed in blocking buffer 3 times for 15 min each, followed by incubation with horseradish peroxidase-conjugated (HRP-conjugated) secondary antibody (Bio-Rad) in 1:10,000 dilution for 1 h at room temperature. Membranes were washed in blocking buffer three times, 15 min each, followed by washing with DPBS. Protein bands were detected using the chemiluminescence detection method of Chemi-Doc (Bio-Rad). Protein bands were quantified by Image lab software and normalized to either HSP70 or β-actin.

### 2.7. Trans-Endothelial/Epithelial Electrical Resistance (TEER)

HUVECs monolayer integrity was analyzed by TEER. Resistance was measured across the monolayer as described previously [20]. Briefly, 0.4 μM transwell inserts (Corning CoStar cooperation) were coated with collagen. HUVECs were plated at a density of 1 × 10^5^ cells/well. TEER was measured as described previously [21,22]. Monolayers were treated with DMSO (control) for 12 h, TNF-α for 12 h, RVX208/JQ1 for 12 h, and pretreatment with RVX208/JQ1 for 12 h followed by TNF-α for 12 h. TEER was measured after 12 h treatments using the epithelial Volt/Ohm meter (EVOM2). Values were normalized against the resistance of a blank well containing only medium and coated with collagen.

### 2.8. Statistical Analysis

GraphPad Prism 5 software was used for statistical analysis of qRT-PCR, Western blot, and TEER data. All experiments were conducted in three or two independent biological replicates and each biological replicate underwent three technical replicates. The mean of all the technical replicates in a biological replicate was used for statistical analysis. Data were analyzed by taking analysis of variance (ANOVA) and Tukey’s or Bonferroni’s post-test, wherever appropriate. All results were plotted in graphs as mean ± standard error of mean (SEM) (* *p* < 0.05, ** *p* < 0.01, *** *p* < 0.001).

## 3. Results

### 3.1. TNF-α Treatment Induces Expression of ProInflammatory Markers

**Figure 1:**
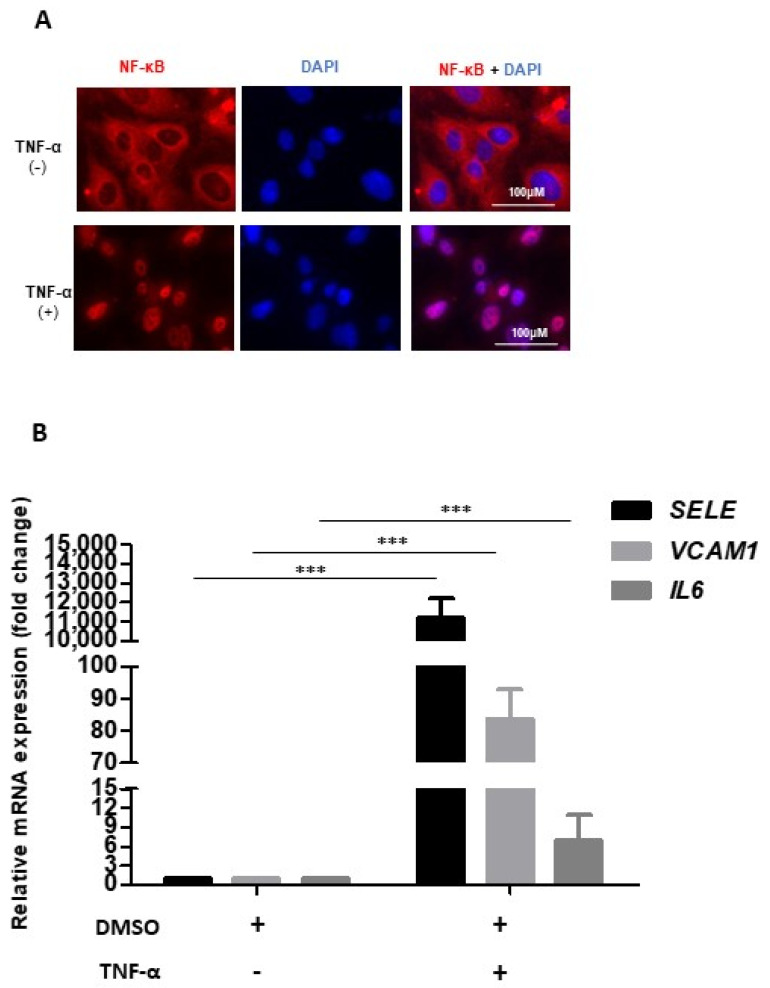
Establishment of inflammatory phenotype in HUVECs. (**A**) Immunofluorescence images showing control (−) and TNF-α treated (+) HUVECs which were stained with anti-NF-κB antibody (red) and nuclei were counter-stained with DAPI (blue). (**B**) Bar graph showing relative mRNA levels of SELE, VCAM-I, and IL6 after normalization to housekeeping gene GAPDH, in DMSO control and TNF-α treated cells. One-way ANOVA with Tukey’s post-test was used; values are mean ± SD of three biological replicates (*** = *p* < 0.0001).

To establish an in vitro inflammatory model, we used human umbilical cord-derived venous vascular endothelial cells (HUVECs), as these resembled the prototypical tool for in vitro EC research. HUVECs were treated with 20 ng/mL TNF-α for two hours, which led to robust translocation of NF-κB into the nucleus (Figure 1A), indicating a sufficient proinflammatory endothelial response. Nuclear NF-κB is known to bind SEs of proinflammatory genes like SELE, VCAM-I, IL6, and IL8 and to upregulate their transcription [7,23]. Indeed, we observed significant increase in the mRNA expression of SELE (11,000 fold), VCAM-I (83 fold), and IL6 (6 fold) in TNF-α treated cells as compared to the controls (Figure 1B). These results indicate that upon TNF-α stimulation expression of E-Selectin (SELE) and vascular cell adhesion molecule-1 (VCAM-1) increases. Both SELE and VCAM-1 are expressed on surface of endothelial cell, promote attachment and transmigration of leukocytes and contribute in the atherosclerotic plaques formation [24]. Furthermore, there was a significant increase in IL-6 mRNA expression, a marker of inflammatory processes [25]. As such, we used TNF-α stimulated HUVECs as a model system for endothelial inflammation.

### 3.2. TNF-α Induces BRD4 Expression in HUVECs

**Figure 2:**
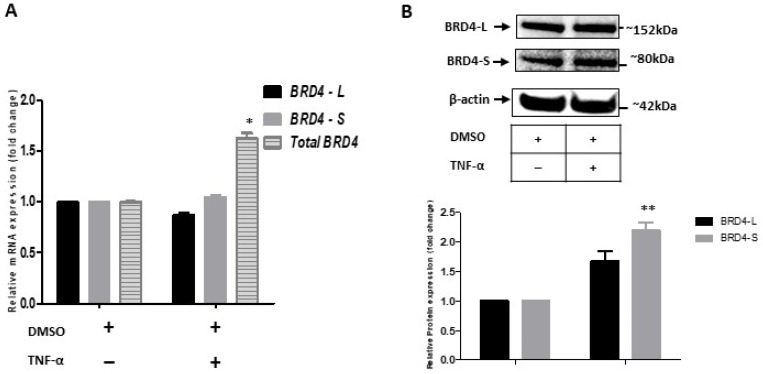
Expression analysis of BRD4 isoforms in HUVECs. (**A**) Bar graph showing relative mRNA levels of BRD4-Total normalized to house-keeping gene GAPDH. BRD4-L and BRD4-S isoforms normalized first to the house-keeping gene and then to BRD4-Total. (**B**) Western blot image and the corresponding relative quantification bar graph showing the protein expression of BRD4-L and BRD4-S. Immunoblot is stripped and reprobed with β-actin antibody. The β-actin (~42kDa) is used as a loading control. The band intensities of BRD4-L and BRD4-S are normalized to β-actin. (**A**,**B**) Two-way ANOVA with Bonferroni’s post-test used; values are mean ± SD of two biological replicates (** = *p* < 0.01 and * = *p* < 0.05).

NF-κB cooperates with BRD4 during an inflammatory response [6,14]. Therefore, we asked whether TNF-α would not only activate NF-κB translocation into the nucleus but also BRD4 expression. BRD4 isoforms have been reported to be differentially expressed in breast and colon cancer [18]. We aimed to determine whether there was any differential expression of BRD4 isoforms in endothelial cells. To analyze relative mRNA expression of BRD4-L and BRD4-S isoforms, isoform specific primers were designed. BRD4-L and BRD4-S expression were first normalized to housekeeping gene GAPDH and then to BRD4-total expression. We checked (BRD4-Total) mRNA expression in our treatment groups. HUVECs were treated with DMSO and TNF-α as described above. mRNA expression analysis revealed a higher expression of total BRD4 (BRD4-Total) in TNF-α treated groups as compared to DMSO control (Figure 2A). At the mRNA level, no significant changes in expression of the transcripts encoding the long and short BRD4 isoform were detectable (Figure 2A). It remained, therefore, an open question whether BRD4 long or BRD4 short isoforms had any exclusive role in expression of proinflammatory genes. However, at the protein level, TNF-α treatment only increased the expression of the short isoform BRD4-S (Figure 2B). Further experiments, especially in animal models, could be useful to elucidate the role of BRD4-S during inflammation. Collectively, the experiments showed that the endothelial inflammation was associated with an increased NF-κB activity and BRD4 expression.

### 3.3. BRD4 Inhibition Reduces mRNA Expression of Inflammatory Marker Genes

Recent reports showed that NF-κB and BRD4 interact and make a complex that binds to SEs of proinflammatory genes during an inflammatory response [6,14]. We, therefore, asked whether BRD4 inhibition would ameliorate the proinflammatory response in a prevention model. To investigate this, we used two small molecule bromodomain inhibitors, JQ1 and RVX208 (Apabetalone).

To our knowledge, this is the first RVX208 study on HUVECs. Therefore, we first checked the appropriate concentration of the RVX208 for experimentation. For this purpose, we treated cells with different concentrations of RVX208: 50-, 100-, 150-, and 200 μM for 4 h. Then, we treated cells with TNF-α for 2 h. Cells were harvested and analyzed for proinflammatory marker SELE. No significant reduction in the SELE expression was observed after 4 h RVX208 pretreatment at any concentration (Supplementary Figure S1A). Next, we pretreated the cells with 60 μM of RVX208 for 12-, 24-, and 48 h, followed by TNF-α treatment for 12 h. We observed significantly reduced expression of SELE after RVX208 pretreatment in all three-time points. Using the same above-mentioned approach, we tested 500 nM and 1 μM concentrations for HUVEC (data not shown) as previously used by Brown et al., 2014 and Wang et al., 2016 [6,26]. We found that 500 nM of JQ1 for 12 h was a sufficient inhibitory dose. Based on this, we selected treatment with 60 µM RVX208 and 500 nm JQ1 for 12 h (as a safe and effective BRD4 inhibitory dose) before stimulation with TNF-α for further experiments.

**Figure 3:**
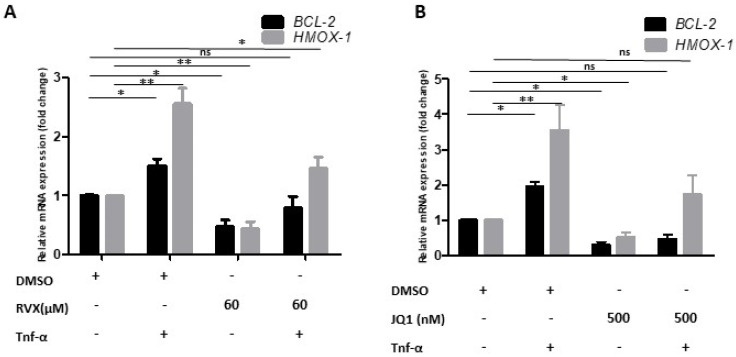
BRD4 inhibitor efficacy. (**A**) Bar graph showing relative mRNA levels of BCL2 and HMOX-1 after normalization to house-keeping gene GAPDH, in DMSO control, TNF-α-only, RVX208, and RVX208, followed by TNF-α treatment (left panel). (**B**) DMSO control, TNF-α-only, JQ1, and JQ followed by TNF-α treatment (right panel). One-way ANOVA with Tukey’s post-test used. Values are mean ± SD of three biological replicates (** = *p* < 0.01, * = *p* < 0.05 and ns = non significant).

The expression of two well-known BRD4 targets, B-cell lymphoma-2 (BCL-2) and heme-oxygenase-1 (HMOX-1), were significantly higher upon TNF-α treatment. Both inhibitors at the above-mentioned concentration could significantly prevent the TNF-α induced BCL-2 and HMOX-1 mRNA expression (Figure 3A,B).

**Figure 4:**
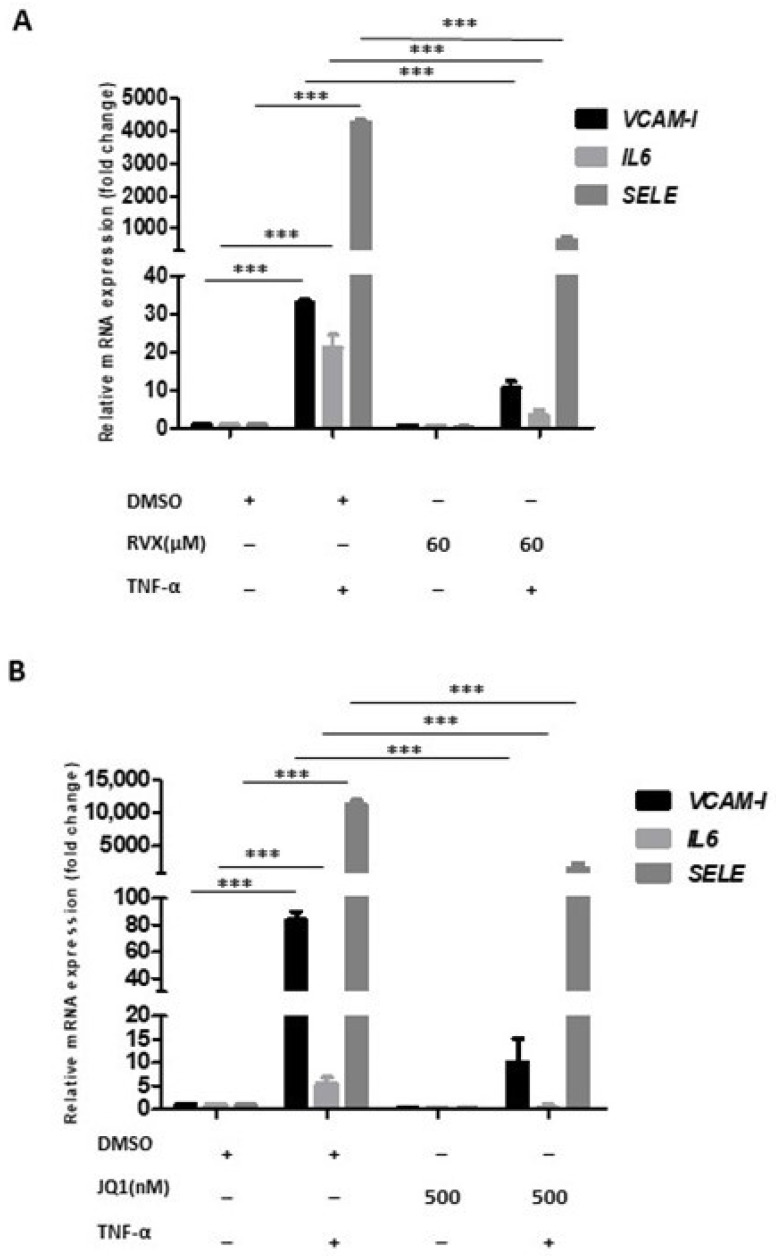
BRD4 inhibition using RVX208 and JQ1. (**A**) bar graph showing relative mRNA levels of inflammatory markers VCAM-I, IL6, and SELE, in HUVECs treated with DMSO, TNF-α-only, RVX208-only, and RVX208-TNF-α (**B**) Bar graph showing relative mRNA levels of inflammatory markers VCAM-I, IL6, and SELE, in HUVECs treated with DMSO, TNF-α-only, JQ1-only and JQ1 + TNF-α. One-way ANOVA with Tukey’s post-test used. Data are shown after normalization to housekeeping gene GAPDH. Values are mean ± SD of three biological replicates (*** = *p* < 0.001).

Next, we investigated how RVX208 pretreatment for 12 h would inhibit TNF-α driven gene induction of VCAM-1, IL6, and SELE. Indeed, RVX208 reduced the expression of VCAM-1, IL6, and SELE 3-, 7- and 6.7-fold, respectively (Figure 4A). Similar results were obtained when using the small molecule bromodomain inhibitor JQ1. Pretreatment with 500 nM of JQ1 could reduce TNF-α driven induction of the proinflammatory markers SELE, VCAM-I, and IL6 by 7-, 8-, and 10-fold, respectively (Figure 4B).

Interestingly, we also observed that both BRD4 inhibitors repressed basal expression (without TNF-α stimulation) of VCAM-1 and IL6 compared to DMSO solvent control. RVX208 treatment reduced basal expression levels of SELE, VCAM-I, and IL6 by 3.7-, 1.4-and 2-fold, respectively. Similarly, JQ1 reduced basal expression levels of SELE, VCAM-I, and IL6 by 7.14-, 3-, and 5-fold, respectively (Supplementary Figure S2).

**Figure 5:**
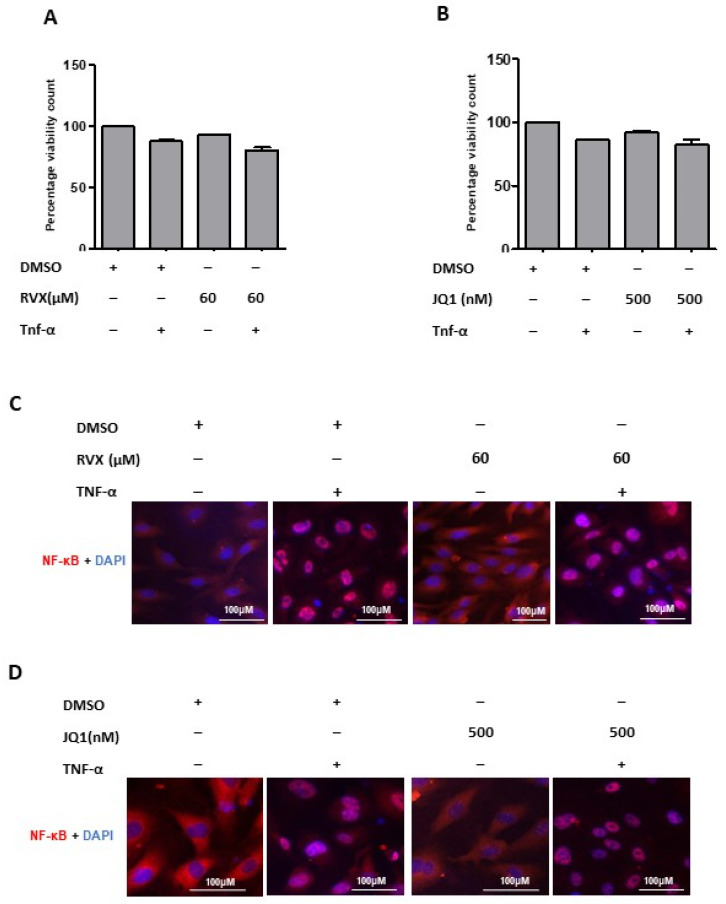
BRD4 inhibitors efficacy. (**A**) Cell viability assay; the bar graph shows percentage cell viability count in treatments with JQ1 after 12 h treatments. (**B**) bar graph shows percentage cell viability count in treatments with RVX208 after 12 h treatments. Inhibition of BRD4 function and target genes expression (**C**,**D**) Immunofluorescence images for the effect of RVX208 and JQ1 on the cellular localization of NF-κB in response to indicated treatments. HUVECs are stained with anti-NF-κB antibody (red) and DAPI (blue). Two-way ANOVA with Bonferroni’s post-test used. Values are mean ± SD of three biological.

To rule out the possibility that the observed effects of both BRD4 inhibitors would be merely the consequence of cell toxicity, we performed cell viability assays. These showed that both treatments, using the same concentrations as the experiments before, had no cytotoxic effects on HUVECs (Figure 5A,B). Additionally, neither BRD4 inhibitor interfered with NF-κB localization, and TNF-α induced NF-κB translocation into the nucleus (Figure 5C,D).

In summary, the data showed that BRD4 inhibition ameliorated proinflammatory gene expression in ECs.

### 3.4. BRD4 Inhibition Protects the Monolayer Integrity of HUVECs during Inflammation

In vascular ECs, TNF-α treatment not only induces proinflammatory gene expression, but also disrupts monolayer integrity and increases its permeability [19,22]. We used TEER measurement to analyze the monolayer integrity of HUVECs during inflammation. HUVECs were grown on collagen-coated transwell membranes to achieve tight monolayer formation [20,27].

**Figure 6:**
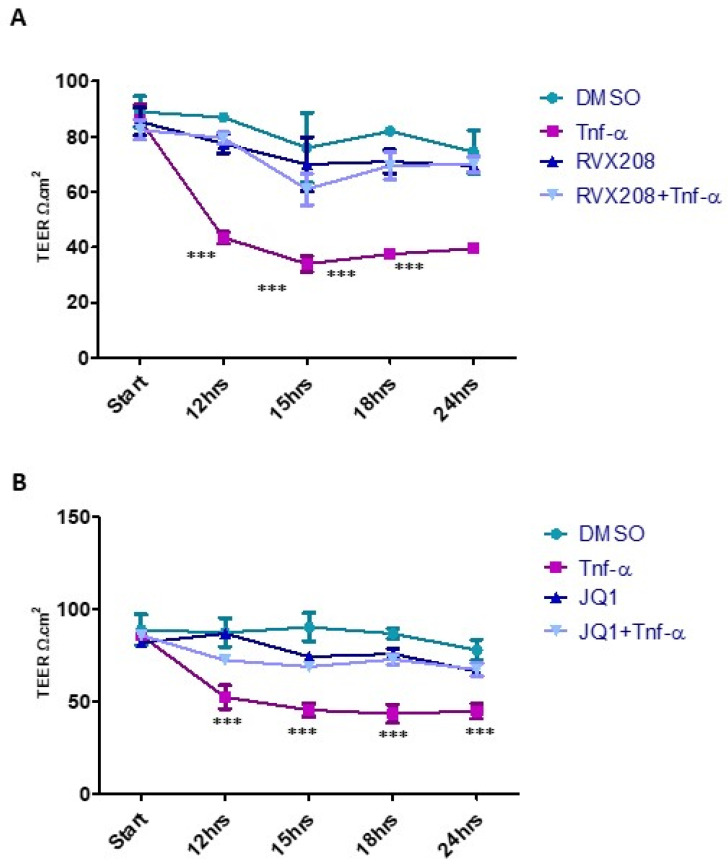
BRD4 inhibition protects monolayer integrity in HUVECs. (**A**,**B**) HUVECs grown on a 0.45 μM transwell membrane for 5 days to establish intact monolayers. HUVECs are treated with DMSO, TNF-α-only, JRVX208/JQ1-only, and JQ1/RVX208, followed by TNF-α. Line graph shows TEER measured in HUVECs monolayer, treated with DMSO and TNF-α for 12 h. Two-way ANOVA with Bonferroni’s post-test used. Values are mean ± SD of three biological replicates (*** = *p* < 0.001).

The endothelial monolayers were first treated with DMSO (control) or TNF-α for 12 h, followed by TEER measurement at different indicated time points over the next 12 h. We observed a significantly reduced electrical resistance of TNF-α treated monolayers, as expected. However, pretreatment with RVX208 (Figure 6A) or JQ1 (Figure 6B), followed by TNF-α treatment, resulted in no significant change in resistance across the monolayer. Hence, the data indicated that BRD4 inhibition by RVX208 or JQ1 played a protective role for endothelial monolayer integrity from proinflammatory stimuli.

### 3.5. BRD4 Inhibition Reduces Midkine Expression during Inflammation

To evaluate whether midkine is involved in the proinflammatory response of endothelial cells, HUVEC monolayers were treated with TNF-α for 2 and 12 h to check the effect on p38MAPK and midkine expression levels during early and late inflammatory responses. The increase in SELE mRNA expression levels confirmed the induction of the inflammatory response (Supplementary Figure S3A). This was associated with increased mRNA expression of p38MAP kinase (p38MAPK) and midkine in TNF-α treated monolayers as compared to control cells (Supplementary Figure S3B). In addition, 6- and 72-h stimulation with TNF-α led to significantly increased midkine mRNA expression (Supplementary Figure S4A). Consistently, Western blot analysis revealed significantly increased midkine protein expression in the supernatant of HUVECs after 36 or 72 h of TNF-α treatment (Supplementary Figure S4B).

**Figure 7:**
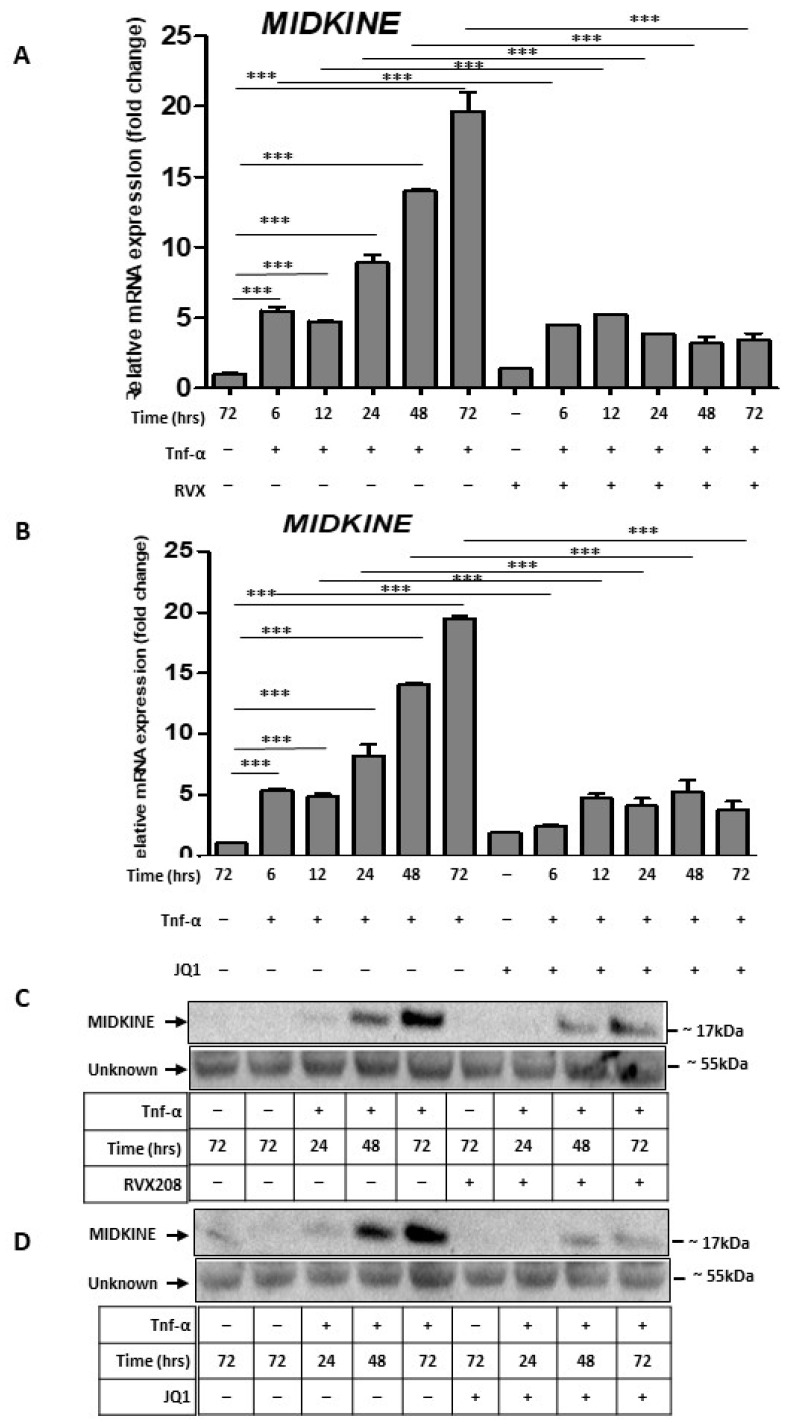
Midkine inhibition in HUVECs with BRD4 inhibitors. (**A**) Bar graph showing relative mRNA levels of midkine in control and TNF-α treatments (6, 12, 24, 48 and 72 h), RVX208 treatment and pretreatment with RVX208 followed by TNF-α (6, 12, 24, 36, 48, and 72 h). (**B**) Bar graph showing relative mRNA levels of midkine in control and TNF-α treatments (6, 12, 24, 48 and 72 h), JQ1 treatment and pretreatment with JQ1 followed by TNF-α (6, 12, 24, 36, 48, and 72 h). One-way ANOVA with Tukey’s post-test used; values are mean ± SD of three biological replicates (*** = *p* < 0.001) Data are shown after normalization to housekeeping gene GAPDH. (**C**,**D**) Western blot image showing the protein expression of midkine in cell supernatant (upper panel) in control, TNF-α treatments (36, 48, and 72 h), and pretreatment with RVX208 (**C**) or JQ1 (**D**) followed by 48 and 72 h of TNF-α treatment. Ponceau staining (lower panel) of the Western blot membrane shows a band of unknown identity at ~55 kDa as a loading control.

Next, we used JQ1 and RVX208 pretreatments for 12 h to inhibit BRD4 in HUVEC monolayers followed by TNF-α from 6 h to 72 h. BRD4 inhibition significantly reduced the midkine expression at mRNA (Figure 7A,B) and protein levels (Figure 7C,D). These results indicated that midkine could be another target of BRD4 during inflammatory response in HUVECs and that inhibition of BRD4 results in decreased expression of midkine.

## 4. Discussion

Atherosclerosis is a major contributor to the development and progression of coronary artery diseases, one of the most frequent causes of death worldwide [1]. Chronic inflammation causes endothelial dysfunction, which leads to the development of atherosclerosis [7]. Translocation of NF-κB, a key mediator of inflammation, drives the expression of inflammatory markers including adhesion molecules (VCAM-I, I-CAM-I, and SELE) and cytokines (IL6 and IL8) [6,7,28]. In our in vitro model, we used HUVECs and treated them with TNF-α to induce inflammation. We observed robust translocation of NF-κB into the nucleus and significantly increased expression of inflammatory markers. This confirmed the establishment of inflammatory phenotype in HUVECs.

BRD4 has been widely studied as an epigenetic modifier. However, its role in the inflammatory response first came into view after it was reported as a NF-κB chromatin partner during inflammation [15]. To gain deeper insight into BRD4 in inflammation, we used a widely-studied BRD4 inhibitor, JQ1 (thienotriazolodiazepine), which binds to bromodomains of BET family proteins BRD2, BRD3, BRD4, and BRDT and inhibits their activity. However, the nonspecificity of JQ1 to all BRD family proteins and its short half-life made it a poor fit for clinical use [29]. Therefore, we compared JQ1 with another inhibitor RVX208 (Apabetalone), an orally active and stable drug which is known for its role in APO-A1 regulation. Apabetalone is already in clinical trials for treatment of different diseases like atherosclerosis, diabetes, Alzheimer’s disease, and chronic kidney disease, and it covalently binds to the BD2-domain of BRD4 [29,30]. Later reports showed that Apo-A-1 was a target of BRD4, but not other BRD proteins [31]. RVX208 isothermal titration calorimetry studies confirmed that RVX208-BRD4 binding affinity was 4-fold higher than with other BRDs [32]. Recent reports showed that RX208 reduced vascular inflammation in vitro as well as in CVDs patients [33]. Keeping in mind the possible off-target effects of any single available small molecules bromodomain inhibitor, we comparatively studied both JQ1 and RVX208 in our investigation.

We confirmed BRD4 inhibition by analyzing two well-known targets of BRD4, BCL-2, and HMOX-1 genes [26,34,35]. Our data showed that both JQ1 and RVX208 pretreated cells had lower expression levels of the proinflammatory markers SELE, VCAM-I, and IL6 upon TNF-α treatment. We also observed that JQ1 or RVX208 treatment alone could reduce the basal expression of SELE, VCAM-I, and IL6. These data indicated that basal level expression of SELE, VCAM-I, and IL6 were at least partially regulated by BRD4, and blocking the activity of BRD4 reduced the expression of these genes, similar to BCL-2 and HMOX-1. This observation was also supported by ChIP-Seq studies of Brown et al. which showed that BRD4 bound to SEs of VCAM-1 and other proinflammatory genes with or without TNF-α stimulation [6].

Endothelial monolayers secrete biologically active substances which contribute to the regulation of the immune system, angiogenesis, and the transport of nutrients [36,37]. Endothelial monolayer disruption via inflammation, oxidative stress, and microbial infection increases permeability and contributes to the development of atherosclerosis and other diseases [38]. We studied in vitro endothelial barrier function. HUVEC monolayers showed reduced TEER and increased permeability when treated with TNF-α, as reported previously [39]. Interestingly, pretreatment with the small molecule BRD4 inhibitors JQ1 and RVX208 protected the endothelial monolayer integrity, along with reducing the inflammatory response. Previously, it was reported that BRD4 inhibition by JQ1 blocked the p21-activated kinase-1 (PAK-1) and reduced VEGF induced permeability [40]. VEGFR2 is an activator of p38MAPK signaling, and blocking of p38MAPK suppresses vascular inflammation. Other cytokines, not only VEGF, can lead to the disruption of an intact endothelial barrier [41]. Among these cytokines, midkine is an interesting candidate as it has been reported to be highly expressed in atherosclerotic plaques and during inflammation [17,42]. NF-κB has been reported as a strong inducer of Midkine and stimulation of cells with TNF-α increased expression of midkine [43]. A noncoding region of the midkine gene contains NF-κB binding sites, indicating that midkine is a target gene of NF-κB. Consistently, inhibition of NF-κB completely inhibited midkine expression [44]. Midkine increased the proliferation of smooth muscle cells, inhibited apoptosis of macrophages, and promoted atherosclerosis plaque formation in mice models [45]. Furthermore, midkine promotes lipid accumulation in macrophages and enhances vascular inflammation, which are key points during atherosclerosis [43]. Moreover, our group has reported that, in human epithelial colorectal adenocarcinoma cells (Caco-2 cells), monolayer permeability is increased by midkine via PI3K pathway activation [18]. We observed significantly higher expression of midkine after TNF-α treatment in HUVECs. Interestingly, BRD4 inhibition by JQ1 or RVX208 also reduced midkine expression. Determining whether the decreased midkine level was directly responsible for better endothelial monolayer integrity during inflammation will require further studies.

## 5. Conclusions

**Figure 8:**
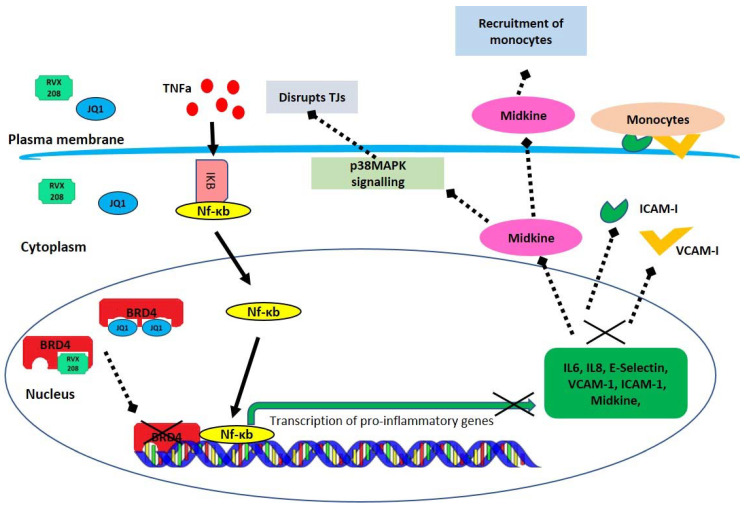
BRD4 inhibition reduces the expression of proinflammatory markers in ECs. Inflammatory stimuli (TNF-α) induces activation and translocation of NF-κB into nucleus. NF-κB, along with BRD4 protein, transcribe proinflammatory markers, including (1) I-CAM, and VCAM-I, which express on ECs monolayer and bind to monocytes from blood and (2) midkine, which contributes to recruitment of monocytes, activates p38MAPK-mediated disruption of tight junctions in ECs, and increases the permeability of ECs monolayer. Small molecular inhibitors of BRD4 bind to BRD4 bromodomains and inhibit its binding to chromatin, which reduces the expression of proinflammatory markers and midkine and protects ECs from dysfunction and tight junction disruption.

In conclusion, our study suggested that BRD4 inhibition with RVX208 or JQ1 coincided with reduced expression of proinflammatory markers, reduced midkine expression, and preserved integrity of endothelial monolayers. Taking reported properties of RVX208 into consideration, our study indicated that RVX208 was an efficient inhibitor of BRD4 and contributed in reducing the inflammatory response and protecting monolayer of ECs, making it a potential therapeutic approach for the treatment of atherosclerosis and other inflammatory diseases (Figure 8). Future studies using animal models will be required to gain deeper insights into these observations.

## Data Availability

Not applicable.

## Supplementary Materials

The following supporting information can be downloaded at: https://www.mdpi.com/1420-3049/27/21/7453/s1, Figure S1: Optimization of Inhibition of BRD4 function; Figure S2: BRD4 inhibition using RVX208 and JQ1. Figure S3: Induction of midkine and p38MAPK expression in HUVECs; Figure S4: Optimization of Midkine expression in HUVEC monolayer during TNF-α treatment; Table S1: Primer sequences for Real time PCR.

## Sample Availability

Samples of the compounds RVX208, JQ1, and TNF-α are available from the authors.
