# Supplementary material for: Small Molecule BRD4 Inhibitors Apabetalone and JQ1 Rescues Endothelial Cells Dysfunction, Protects Monolayer Integrity and Reduces Midkine Expression
Source: Molecules. 2022 Nov 2;27(21):7453. doi: 10.3390/molecules27217453 (PMC9692972; doi:10.3390/molecules27217453)
Supplement: Supplementary file 1 [file molecules-27-07453-s001.zip › molecules-1923914-supplementary.pdf]

molecules

MDPI

Supplementary Figures

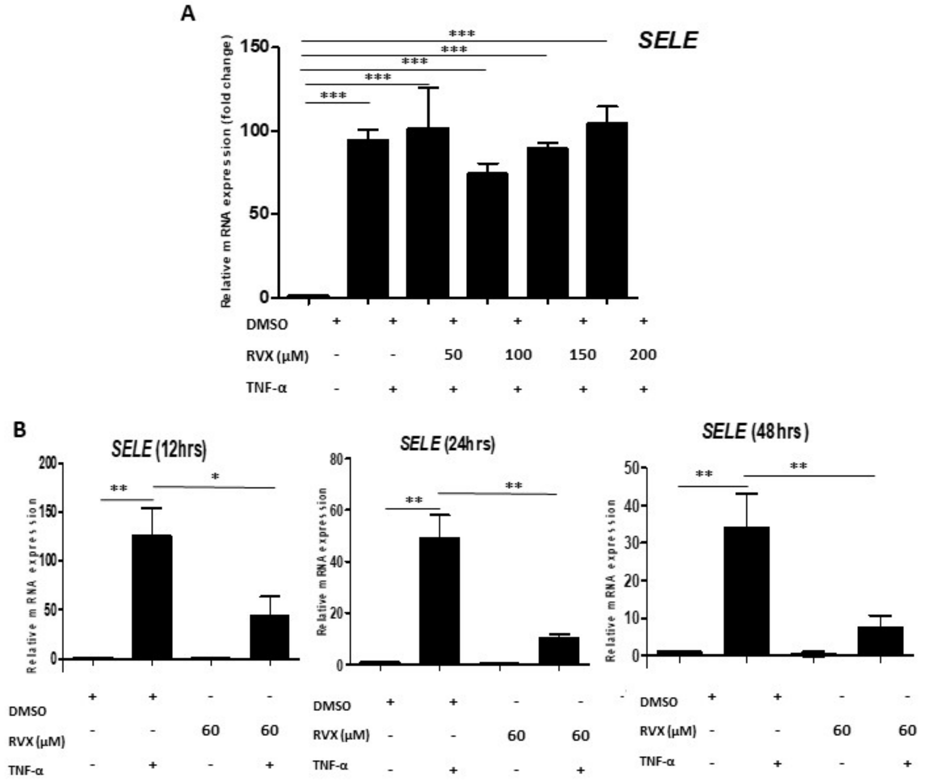

Figure S1. Optimization of Inhibition of BRD4 function. **A**. bar graph showing relative mRNA levels of SELE after normalization to house-keeping gene GAPDH in DMSO (control), TNF-α treatment, RVX208 (50-, 100-, 150-, and 200 μM) for four hours followed by TNF-α treatment. **B**. bar graph showing relative mRNA levels of SELE after normalization to house-keeping gene GAPDH, in DMSO control, TNF-α-only, RVX208-only 60 μM (12 hrs to 48 hrs), and RVX208 (60 μM) followed by TNF-α treatment. A-B. One-way ANOVA with Tukey's post-test was used and treated samples were compared with DMSO treated samples, and values are mean ± SD of three biological replicates (***= p< 0.001** = p<0.01, and * = p<0.05)).

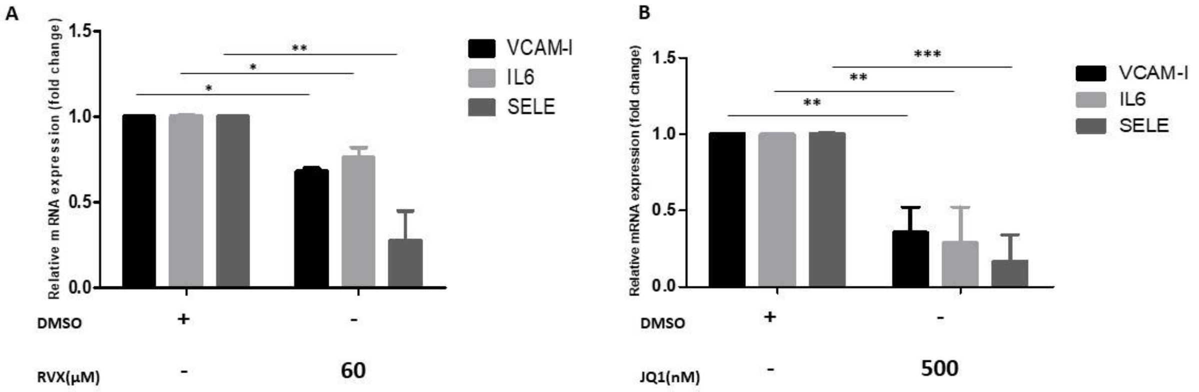

Figure S2. BRD4 inhibition using RVX208 and JQ1. **A**. bar graph showing relative mRNA levels of inflammatory markers VCAM-I, IL6, and SELE, in HUVECs, treated with DMSO, and RVX208-only **B**. Bar graph showing relative mRNA levels of inflammatory markers VCAM-I, IL6, and SELE, in HUVECs, treated with DMSO, and JQ1-only. One-way ANOVA with Tukey's post-test was used. Data are shown after normalization to housekeeping gene GAPDH. Values are mean ± SD of three biological replicates (***= p< 0.001** = p<0.01, and * = p<0.05).

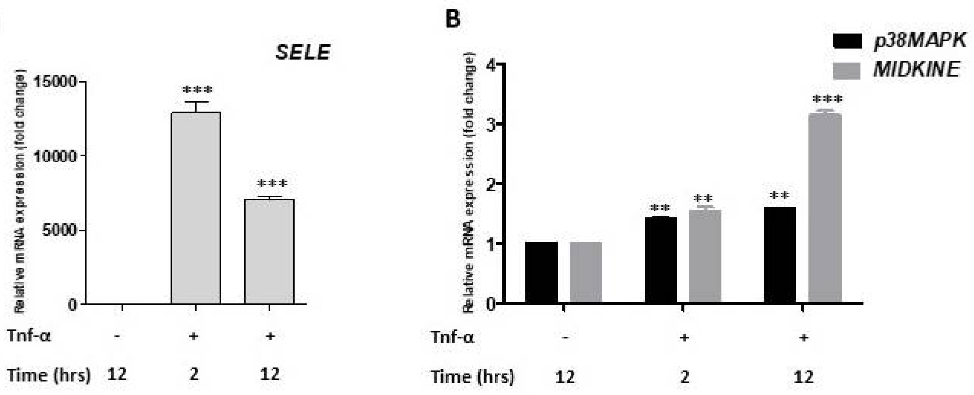

Figure S3. Induction of midkine and p38MAPK expression in HUVECs. **A**, Bar graph showing mRNA levels of SELE after normalization to house-keeping gene GAPDH in Control (non-treated) and in TNF-α treated (2hrs and 12hrs) monolayers. **B**, Bar graph showing mRNA levels of p38MAPK and midkine after normalization to house-keeping gene GAPDH in non-treated control and TNF-α treated (2 hrs and 12 hrs) monolayers. One-way ANOVA with Tukey's post-test was used, and values are mean ± SD of two biological replicates and asterisks indicate statistical significance of *** p< 0.001 and, ** p<0.01.

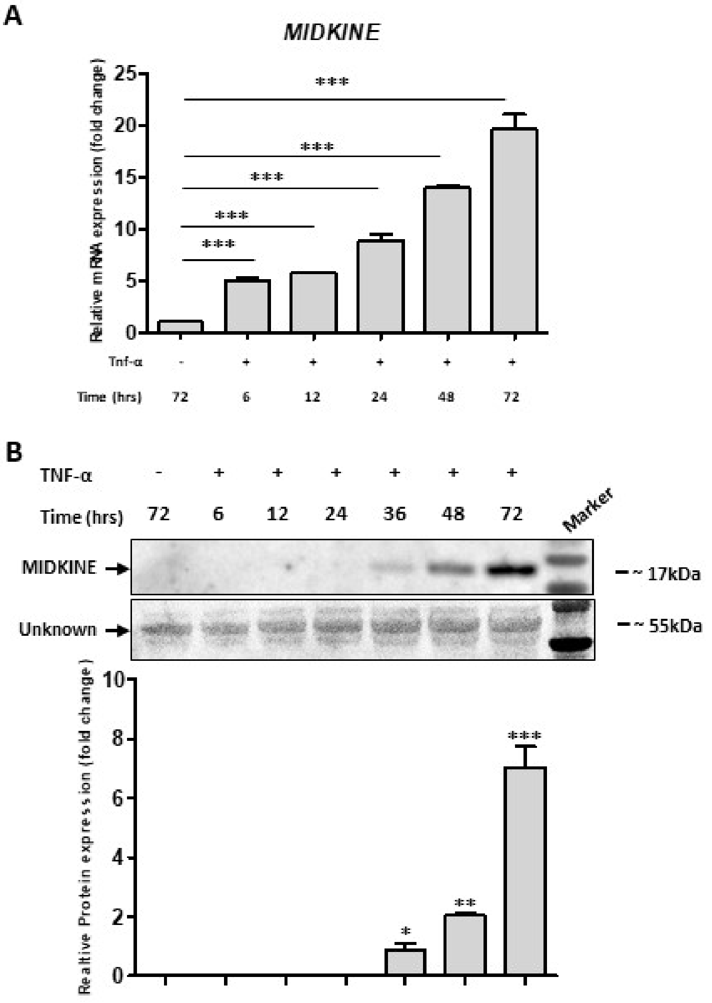

Figure S4. Optimization of Midkine expression in HUVEC monolayer during TNF-α treatment: **A**. Bar graph showing relative mRNA levels of midkine after normalization to house-keeping gene, GAPDH, in control and after different times of TNF-α treatments (6-, 12-, 24-, 48- and 72-hrs). **B**. Western blot image (upper panel) and the corresponding relative quantification bar graph showing

the midkine protein expression in cell supernatant (lower panel) in control and TNF-α treatments (6-, 12-, 24-, 36-, 48-, and 72hrs). Ponceau staining of the Western blot membrane shows a band of unknown identity at ~55 kDa as a loading control. One-way ANOVA with Tukey's post-test was used, and values are mean ± SD of three biological replicates (*** = p< 0.001, ** = p<0.01 and * = p<0.05).

Table S1. Primer sequences for Real time PCR.

No.	Gene	Primer sequence
1	GAPDH	forward primer 5′TGGGTGTGAACCATGAGAAGTA3′
		reverse primer 5′GAGTCCTTCCACGATACCAAAG3′
2	SELE	forward primer 5′CTCTCCCTCCTGACATTAGCAC3′
		reverse primer 5′AGGCTTTTGGTAGCTTCCATCT3′
3	VCAM-I	forward primer 5′GGAAAAACAGAAAAGAGGTGGA3′
		reverse primer 5′GCCCATGACACTACATGTCAAC3′
4	IL6	forward primer 5′AGTGAGGAACAAGCCAGAGC3′
		reverse primer 5`GTCAGGGGTGGTTATTGCAT3′
5	BRD4-total	forward primer 5′TCCAACCCTAACAAGCCCAA3′
		reverse primer 5`GAAAGGCCATGCAAACTGGT3′
6	BRD4-Short isoform	forward primer 5′TCCTCCAAGATGAAGGGCTT3′
		reverse primer 5′AGCTTGCTGGGAAGGAATCT3′
7	BRD4-Long isoform	forward primer 5′AGCGAAGACTCCGAAACAGA3′
		reverse primer 5′TCTGCTGATGGTGGTGATGA3′
8	MIDKINE	forward primer 5′ACCAGTGCCTTCTGTCTGCT3′
		reverse primer 5′ATTGTGGGGAAGAACAAAAGC3′
9	P38MAPK	forward primer 5′TGGTACTGAGCAAAGTAGGCA3′
		reverse primer 5′ TGGGAAATGCAGGGAGTTCT3′
